# Cognitive Mechanisms of Predictive Processing in Chinese Reading: An Eye-Movement Analysis Based on the Ex-Gaussian Distribution

**DOI:** 10.3390/jemr19030054

**Published:** 2026-05-15

**Authors:** Wen Tong, Xiaojiao Li, Yingdi Liu, Zhifang Liu

**Affiliations:** 1 School of Psychology, Shanxi Normal University, Taiyuan 030031, China; twaaa@126.com; 2 College of Jinghengyi Education, Hangzhou Normal University, Hangzhou 311121, China; 2025112004211@stu.hznu.edu.cn (X.L.); 15294910487@163.com (Y.L.)

**Keywords:** contextual predictability, Ex-Gaussian distribution, eye-movement tracking, Chinese reading, conflict resolution

## Abstract

This study employed the Ex-Gaussian distribution model to analyse eye-tracking data, to elucidate the cognitive mechanisms underlying predictive processing during Chinese reading. Using a single-factor, two-level within-subjects design (contextual predictability: high vs. low), data from 32 adult readers were analysed across the pre-target and target word regions. The results revealed that predictive reading follows a three-stage cognitive model. In the expectation generation stage (pre-target region), a significant negative τ effect indicated resource pre-allocation driven by strong contextual constraints, thereby facilitating the construction of predictive lexical representations. In the verification and integration stage (target word region), a significant negative μ effect in the later measurement window indicated that successful prediction–input matching accelerated lexical identification and enhanced integration efficiency; the σ parameter did not reach significance in either measurement window. In the conflict resolution stage (pre-target and target word regions), a significant positive τ effect indicated that verification failure triggered lexical activation competition at the target word, driving regressive fixations to the pre-target region for contextual reanalysis; conflict resolution costs were markedly higher under the low-predictability condition, owing to the absence of a dominant activation anchor. These findings suggest that contextual predictability influences reading through a dual mechanism: the μ parameter modulates the automatic processing speed of lexical identification, whereas the τ parameter regulates the cognitive control processes underlying expectation generation and conflict resolution. Together, these results provide empirical support for the integration of predictive coding theory and cognitive control frameworks.

## 1. Introduction

During reading, readers can exploit contextual information to predict upcoming words, and this predictive processing is widely regarded as a key mechanism underlying efficient reading. A substantial body of research has demonstrated that high-predictability contexts facilitate the processing of target words, as evidenced by shorter fixation durations and reduced N400 amplitude in electroencephalographic recordings [1,2]. This “predictability benefit” not only highlights the anticipatory nature of language comprehension but also raises fundamental questions about the nature of the underlying cognitive processes, their temporal dynamics, and the mechanistic distinctions across different levels of predictability.

To address the question of the cognitive mechanisms underlying predictive processing, several competing theoretical perspectives have emerged in the literature. Proponents of the automaticity view contend that predictive processing constitutes a rapid, associative activation process intrinsic to the language system, whereby lexical representations consistent with the current context are spontaneously pre-activated within an extremely narrow time window—typically 100–200 milliseconds—without requiring additional cognitive effort or attentional resource allocation [3,4]. In contrast, the controlled processing hypothesis holds that predictability effects rely on the recruitment of the executive control system: readers must actively engage working memory resources to maintain and manipulate contextual information and employ top-down attentional mechanisms to generate and verify predictions about upcoming words. This process is cognitively demanding and is substantially modulated by individual differences [5]. Recent research has further demonstrated that these two mechanisms may not be mutually exclusive; rather, they likely interact dynamically as a function of task conditions. Specifically, automatic activation predominates in highly predictable contexts, whereas controlled processing assumes greater importance in complex or ambiguous linguistic environments [4].

Although existing theoretical frameworks have provided important perspectives for understanding predictive processing, they remain limited in their ability to fully explain the cognitive mechanisms underlying predictability effects. Predictive Coding Theory posits that the brain comprehends incoming information by generating predictions and continuously updating prediction errors [6,7]. While this framework effectively accounts for the neural basis of predictability effects, it provides little insight into the cognitive processes involved in updating or revising failed predictions. Similarly, Cognitive Control Theory emphasises the critical role of executive functions in language processing [8] yet offers limited insight into the temporal dynamics of control processes during predictive processing.

These theoretical limitations largely reflect methodological constraints inherent in conventional research approaches. Most studies on predictive processing have relied on aggregate measures—such as mean response time, total reading time, or mean amplitude of event-related potentials—and conducted statistical analyses under the assumption of normality. Although such approaches are capable of detecting the presence and magnitude of predictability effects, they are ill-suited to decomposing the underlying cognitive components. In particular, they cannot readily distinguish the contributions of rapid automatic processing from those of deliberate controlled processing, nor can they reveal the multiple cognitive mechanisms operative during predictive processing or the temporal ordering among them. Critically, the prevailing reliance on binary predictability contrasts (predictable vs. unpredictable) further limits theoretical progress: by collapsing across what may be temporally distinct cognitive sub-stages of predictive processing—spanning pre-target expectation construction through to post-target integration and potential reanalysis—mean-based analyses obscure whether the same or qualitatively different mechanisms drive predictability effects at each stage. An approach sensitive to the full distributional shape of reading time data, and capable of isolating stage-specific cognitive signatures, is therefore needed.

Ex-Gaussian distributional analysis offers a powerful methodological approach for decomposing response time distributions into distinct cognitive components. The Ex-Gaussian distribution—defined as the convolution of a normal distribution and an exponential distribution—fits the right-skewed distribution of response time data well. This distribution is characterised by three parameters. The μ parameter represents the mean of the normal component and primarily reflects the central tendency and baseline speed of cognitive processing. The σ parameter represents the standard deviation of the normal component and captures the variability in and consistency of processing. The τ parameter corresponds to the scale parameter of the exponential component and selectively indexes the elongation of the right tail of the distribution, reflecting slow, controlled processes that require additional cognitive resources [9,10]. Whereas the μ and σ parameters predominantly reflect rapid, relatively automatic cognitive processing, the τ parameter is closely associated with resource-demanding processes such as attentional control, working memory updating, error monitoring, and strategic processing [11].

Within the field of language processing, Ex-Gaussian analysis has proven particularly informative. Staub et al. [12] showed that word frequency effects were primarily reflected in the τ parameter, indicating that the processing of low-frequency words requires additional cognitive control resources. Converging evidence comes from a study of lexical predictability by Staub [13], in which words that were difficult to predict selectively affected the τ parameter rather than the μ or σ parameters, supporting the critical role of controlled processing in handling unpredictable information. In syntactic processing research, Balota and Yap [9] similarly found that syntactic complexity was predominantly associated with increases in the τ parameter. Collectively, these findings suggest that the τ parameter serves as a particularly sensitive index of controlled processing demands across a range of language processing contexts.

Despite the methodological utility that Ex-Gaussian analysis has demonstrated in language processing research, its systematic application to the study of contextual predictability remains limited, with an especially notable absence of empirical investigation in the context of Chinese reading. As a logographic writing system, Chinese possesses orthographic and syntactic properties that place particularly strong demands on predictive processing during reading. At the orthographic level, Chinese lacks inter-word spaces, requiring readers to rely on semantic and syntactic information for real-time word segmentation [14,15]. Moreover, the highly complex correspondence between Chinese characters and words—whereby a single character may function as an independent word or combine with other characters to form multi-character words—renders lexical identification heavily dependent on contextual constraints [16]. At the syntactic level, Chinese lacks rich morphological inflexions, with grammatical relations expressed primarily through word order and context; consequently, identical character sequences may assume entirely different grammatical functions and semantic roles across different contexts [17,18]. These features collectively result in a heightened reliance on contextual prediction during Chinese reading. In high-predictability contexts, readers can exploit contextual information to facilitate rapid word segmentation and lexical identification. In low-predictability contexts, however, prediction failure triggers reanalysis of word segmentation, ambiguity resolution, and revision of the syntactic structure—processes that may impose particularly high cognitive control demands in Chinese reading.

The present study addresses the gap identified above. Eye-movement measures provide a continuous, online record of cognitive processing with millisecond-level temporal resolution, with distinct measures reflecting different stages of reading: first fixation duration (FFD) primarily indexes early lexical identification and initial semantic activation; gaze duration (GD) reflects lexical identification as well as early syntactic and semantic integration; and total reading time (TRT) captures the complete processing sequence, including regressive fixations [19,20]. By applying Ex-Gaussian analysis to these measures across both the pre-target region and the target word region in Chinese sentence reading, the present study aims to move beyond the binary predictability contrast and reveal the temporal architecture of predictive processing: specifically, to determine which cognitive sub-mechanisms—automatic or controlled—are engaged at each processing stage and in what order they unfold. This question has not been directly addressed in prior research, and its answer carries implications for both theoretical accounts of predictive language processing and the development of more sensitive analytical tools for reading research.

We hypothesise that if the predictability effect is primarily driven by automatic processing, it should be reflected in the μ and σ parameters of fixation duration distributions. Conversely, if controlled processing plays a dominant role, the effect should be predominantly captured by the τ parameter. The present study tests these hypotheses by examining how predictability effects manifest across distinct processing stages: specifically, we analyse early processing windows (FFD and GD) and a later processing window (TRT) separately for both the pre-target region and the target word region, allowing us to trace the temporal dynamics of predictability effects as they unfold across different spatial locations and processing depths.

## 2. Materials and Methods

### 2.1. Participants

We estimated the required sample size using G*Power (version 3.1), specifying an effect size of *d* = 0.5, α = 0.05, and power = 0.80. This analysis indicated a minimum required sample size of 27 participants. To ensure adequate statistical power after anticipated data exclusions (typically 10–15% in eye-tracking research), we recruited 32 native Mandarin-speaking university students (18 women, 14 men; mean age = 22.3 years, *SD* = 2.1). All participants were native Mandarin-speaking university students with normal or corrected-to-normal visual acuity and no history of reading disability; all were right-handed. Participation was voluntary, and all participants provided written informed consent before the experiment began. Participants received monetary compensation for their participation. The experimental protocol was approved by the Institutional Ethics Committee.

### 2.2. Experimental Design

The present study employed a within-subjects design with target word predictability as a two-level factor (high vs. low predictability). The dependent variables were the following eye-movement measures, extracted from predefined regions of interest (ROIs): first fixation duration (FFD), gaze duration (GD), and total reading time (TRT).

### 2.3. Materials

#### 2.3.1. Target Word Selection

A total of 240 two-character nouns were drawn from the SUBTLEX-CH corpus [21] and used as target words. Word frequency ranged from 0.06 to 1110.19 occurrences per million (*M* = 43.12, *SD* = 109.82), and stroke count (summed across both characters) ranged from 7 to 27 (*M* = 15.55, *SD* = 3.82).

#### 2.3.2. Sentence Construction

We constructed two sentence contexts for each target word (one per predictability condition; see Table 1). Sentence length ranged from 11 to 23 Chinese characters. In all sentences, the word immediately preceding the target (hereafter the pretarget word) was a two-character word; pretarget words were matched across the high- and low-predictability conditions on character frequency and stroke count (all *p*s > 0.05; see Table 2).

#### 2.3.3. Predictability Norming

Twenty university students who did not participate in the main experiment completed a cloze task to establish predictability norms for the stimulus sentences. Participants were presented with the sentence frame up to but not including the target word and were asked to complete the sentence with the first word that came to mind. Cloze probabilities were significantly higher in the high-predictability condition (*M* = 84.35%, *SD* = 7.21%) than in the low-predictability condition (*M* = 1.70%, *SD* = 1.53%), *t*(239) = −72.24, *p* < 0.001.

### 2.4. Apparatus

Eye movements were recorded using an EyeLink 1000 eye tracker (SR Research, Mississauga, ON, Canada) at a sampling rate of 1000 Hz using monocular recording of the dominant eye, with a spatial resolution of 0.01°. Stimuli were displayed on a 19-inch monitor (screen resolution: 1024 × 768 pixels; refresh rate: 75 Hz). Viewing distance was maintained at 60 cm using a chinrest. All experimental sentences were presented in a single line in 20-point Song typeface (宋体), with each Chinese character subtending approximately 1° of visual angle.

### 2.5. Procedure

Prior to the experiment, participants read the task instructions and completed a 3-point horizontal calibration and validation procedure; calibration was accepted only when the average error across all validation points was less than 0.5°; otherwise, calibration was repeated. A drift correction was performed at the beginning of each trial via fixation on a dot positioned at the sentence onset location. Participants completed 10 practice trials prior to the main experiment. The main experiment consisted of 480 trials, constructed from 240 target words each appearing in both a high-predictability and a low-predictability sentence frame; all participants read all 480 sentences in a fully within-participant design, with no list assignment or filler sentences. Trials were divided into 6 blocks of 80 trials each, with rest breaks provided between blocks, and presented in a randomised order generated independently for each participant. Each trial began with a fixation cross presented for 800 ms. Participants then triggered sentence presentation by pressing the spacebar and read the sentence silently. Upon completing reading, they pressed the downward arrow key (↓) to proceed to the next trial. To monitor reading comprehension, a yes/no comprehension question followed a randomly selected 25% of trials, to which participants responded by pressing the left arrow key (←) for incorrect and the right arrow key (→) for correct; each participant’s comprehension score was calculated as the proportion of correct responses across all such questions. The total duration of the experiment was approximately 45 min.

## 3. Results

### 3.1. Data Preprocessing

Analyses focused on FFD, GD, and TRT in two predefined regions of interest: the pre-target region and the target word region. Data cleaning was conducted at two levels. At the participant level, any participant whose comprehension question accuracy fell below 80% was to be excluded from all analyses; however, all 32 participants met this criterion and were therefore retained in the final sample. At the trial level, fixations with durations shorter than 80 ms or longer than 1200 ms were removed as outliers, and remaining values exceeding ±3 standard deviations from the condition-specific mean were trimmed as statistical outliers. These trial-level procedures retained 92.3% of the total fixation data.

### 3.2. Analysis of Eye-Movement Measures for the Target Word and Pre-Target Regions

Eye-movement measures for the pre-target and target word regions under each predictability condition are presented in Table 3. Paired-samples *t*-tests were conducted for each eye-movement measure in both regions (low predictability minus high predictability). In the pre-target region, FFD did not differ significantly between conditions, *t*(31) = −1.646, *p* = 0.110, *d* = −0.291; GD was significantly longer under the high-predictability condition than under the low-predictability condition, *t*(31) = −4.251, *p* < 0.001, *d* = −0.751; and TRT did not reach statistical significance, *t*(31) = 1.954, *p* = 0.060, *d* = 0.345. In the target word region, FFD was significantly longer under the low-predictability condition than under the high-predictability condition, *t*(31) = 2.909, *p* = 0.007, *d* = 0.514; GD was similarly longer under the low-predictability condition, *t*(31) = 2.739, *p* = 0.010, *d* = 0.484; and TRT was significantly longer under the low-predictability condition, *t*(31) = 5.502, *p* < 0.001, *d* = 0.973.

### 3.3. Ex-Gaussian Parameter Analysis

An Ex-Gaussian distribution was fitted to each participant’s fixation duration data for each condition using the retimes package (version 0.1-2) [22] in R (version 4.4.1) [23]. This procedure yielded estimates of three parameters: μ (mean of the normal component), σ (standard deviation of the normal component), and τ (mean of the exponential component). Parameters were estimated via maximum likelihood estimation (MLE), with a convergence criterion of 1 × 10^−6^ (change in log-likelihood) and a maximum of 1000 iterations. Goodness of fit was evaluated using log-likelihood values, AIC/BIC criteria, the Kolmogorov–Smirnov test, and R^2^; the mean R^2^ across participants exceeded 0.92, indicating adequate model fit. Following parameter extraction at the participant level, linear mixed-effects models [24] were fitted separately for each of the three parameters, with predictability condition as a fixed effect and by-participant random intercepts and random slopes for predictability condition. Degrees of freedom were estimated using the Satterthwaite approximation [25], and all pairwise comparisons were corrected for multiple testing using the false discovery rate (FDR) procedure [26]. Full results are presented in Table 4.

## 4. Discussion

The present study employed both conventional eye-movement measures and Ex-Gaussian distributional analysis to elucidate the mechanisms by which contextual predictability influences eye-movement patterns during Chinese reading. With respect to the conventional eye-movement measures, gaze duration in the pre-target region was longer under the high-predictability condition than under the low-predictability condition, suggesting that readers may allocate cognitive resources to predictive processing prior to the appearance of the target word. This pattern may reflect the prospective nature of predictive reading. In the target word region, all three eye-movement measures yielded significantly shorter fixation durations under the high-predictability condition relative to the low-predictability condition, suggesting a facilitative effect of expectation verification on lexical identification.

Further Ex-Gaussian distributional analyses provided more nuanced insights into the locus of the predictability effect. In the pre-target region, condition differences were predominantly reflected in the τ parameter: τ approached significance in the FFD window (*p* = 0.064) and reached significance in both the GD (*p* = 0.031) and TRT windows (*p* = 0.022), whereas μ and σ remained unaffected across all windows. In the target word region, a dissociation among the three parameters was observed across eye-movement measures: within the FFD and GD windows, μ reached significance and σ approached significance, while τ was non-significant; within the TRT window, τ reached its maximum effect size (L − H = +19.81 ms, *p* = 0.001), while μ and σ were non-significant. Notably, the τ difference in the pre-target GD window was negative in direction (L − H = −7.87 ms), consistent with the direction of the τ effect during the expectation-building phase. Based on the pattern of parameter effects across the three eye-movement measures, we propose a three-stage account of the cognitive mechanisms underlying the predictability effect: (1) an expectation-building stage, (2) an expectation-verification and integration stage, and (3) a conflict-resolution stage.

### 4.1. A Cognitive Mechanism Model of Predictive Reading

**Figure 1:**
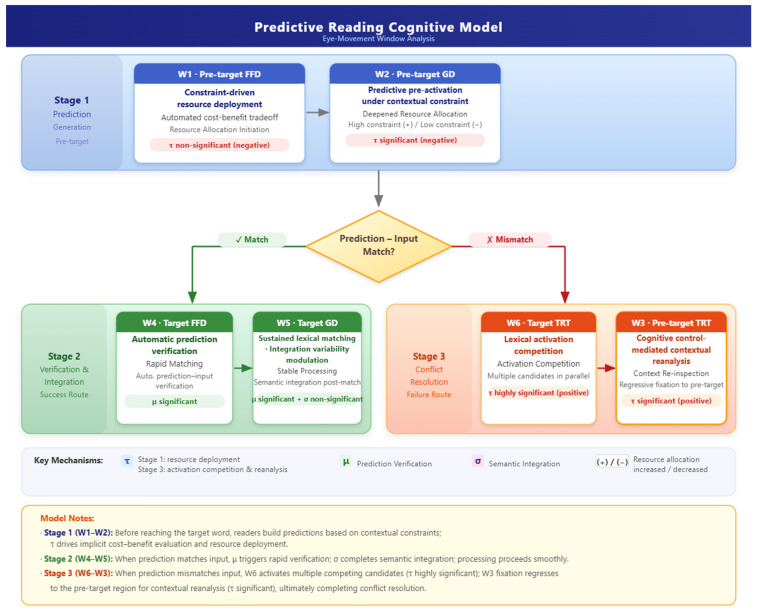
A three-stage model of predictive processing in reading.

Drawing on the Ex-Gaussian analyses, the predictability effect in reading can be conceptualised as encompassing three core processing stages: expectation generation, expectation verification and integration, and conflict resolution. Each stage appears to be characterised by a distinct pattern of parameter effects and may be associated with different processing pathways under high- versus low-predictability conditions. A common organising principle is hypothesised to underlie all three stages: the dynamic interplay between automatic and controlled processing [27,28]. Automatic processing—hypothesised to manifest as rapid spreading activation and lexical representation matching driven by contextual constraints—may be reflected in the μ and σ parameters. Controlled processing, by contrast—potentially manifesting as top-down expectation construction and conflict repair, both of which are regulated by cognitive resources—could be indexed by the τ parameter. Huettig [29] explicitly proposed that predictive language processing involves two dissociable routes: a “dumb route” based on associative activation—corresponding to automatic processing—and a “smart route” based on combinatorial processing—corresponding to controlled processing. The relative contribution of each route at different processing stages is hypothesised to shape the cognitive signature of the predictability effect. The findings supporting the three-stage model proposed in the present study provide temporal patterns broadly consistent with this dual-route hypothesis (see Figure 1).

#### 4.1.1. Stage 1: Expectation Generation (Pre-Target Region, W1–W2)

At this stage, the data tell a strikingly selective story: only τ was sensitive to the predictability manipulation, while μ and σ remained entirely unaffected.

The expectation generation stage occurs in the pre-target region, spanning two measurement windows: W1 (FFD) and W2 (GD). Neither μ nor σ differed significantly across conditions in W1–W2, whereas τ did not reach significance in W1 but reached significance in W2. This pattern suggests that condition differences at the current stage are predominantly carried by τ, is consistent with a top-down process in which the cognitive system may draw on contextual cues to construct lexical predictions.

The predominance of the τ effect at this stage is consistent with theoretical accounts emphasising the role of controlled processing in predictive resource allocation. Kuperberg and Jaeger [4] argued that the degree of pre-activation is determined by its “expected utility”—that is, the reader’s implicit evaluation of the trade-off between predictive gains and processing costs governs the level of controlled resources invested. The significant τ effect observed in W2 is consistent with such constraint-driven resource deployment: under the high-predictability condition, strong contextual constraints provide an unambiguous signal of predictive gain, potentially leading the system to commit greater controlled resources; under the low-predictability condition, the predictive gain signal is weak, and resource investment is correspondingly reduced. As contextual processing deepens into the GD window (W2), accumulated constraint information drives predictive pre-activation under contextual constraint, suggesting that expectation construction may be completed prior to the arrival of the target word. The negative direction of the τ difference in GD (i.e., τ was larger under the high-predictability condition than under the low-predictability condition) may reflect the flexible, graded regulation of controlled processing across these two sub-stages of resource deployment and pre-activation.

Although τ dominated the effect pattern at this stage, the non-significance of μ and σ suggests that automatic activation may have remained at a sub-threshold level—contextually driven spreading activation may have been initiated but may not yet reached the magnitude required to produce measurable behavioural effects. Neely [30], through a series of semantic priming experiments, provided evidence suggesting that automatic spreading activation and controlled expectancy mechanisms may be dissociable, such that automatic activation can operate independently at short SOAs, though its effect size is constrained by the range of activation spread. Lau et al. [31] further provided neural-level evidence for sub-threshold automatic activation, identifying automatic semantic facilitation effects in the anterior temporal cortex through combined fMRI and MEG studies. Together, these findings suggest that at this stage, controlled processing may establish the conditions for subsequent automatic processing: the allocation of controlled resources may determine the depth and precision of expectation construction, thereby setting the stage for efficient automatic verification in subsequent processing stages.

Chang et al. [32] provided evidence for graded predictive pre-activation during Chinese sentence reading, with more pronounced predictability effects under strong contextual constraint conditions. This graded pattern of controlled resource investment is consistent with a particular form of expectation construction costs while also implying a potential cost: the more precisely a lexical representation is pre-activated, the greater the potential processing conflict when verification fails.

In plain terms: before readers even reach the critical word, they appear to be actively drawing on sentence context to predict what comes next—and the stronger the context, the more cognitive resources they appear to invest in building that prediction.

#### 4.1.2. Stage 2: Expectation Verification and Integration (Target Word Region—Success Route, W4–W5)

Whereas Stage 1 was driven entirely by τ, Stage 2 presents the opposite pattern: τ falls silent, and it is μ and σ that now carry the predictability effect—which could represent a parameter-level signature of the transition from deliberate preparation to fluent, automatic execution.

The expectation verification and integration stage unfolds in the target word region, spanning measurement windows W4 and W5. This stage is hypothesised to occur primarily when the pre-activated lexical representation successfully matches the incoming input, and comprises two sequential sub-mechanisms: a verification sub-process potentially indexed by μ and an integration sub-process potentially indexed by σ.

In marked contrast to Stage 1, the parameter effects at this stage are predominantly carried by μ and σ, suggesting a potential dominance of automatic processing in expectation verification and integration. The μ parameter exhibited a significant predictability facilitation effect in W4 (smaller μ values under the high-predictability condition), which may reflect a rapid-matching verification process in which the pre-activated lexical representation is compared against the actual input. This process is consistent with the defining characteristics of automatic processing—rapid and requiring minimal attentional resources. Pickering and Garrod [33] proposed that prediction in language comprehension relies on a forward model, wherein the comparison between predicted and actual input constitutes a fast, automatic process. Upon successful verification, processing appears to transition into the integration sub-stage, as suggested by the continued significant effect of μ in W5. The σ parameter did not reach significance in W5 (*p* > 0.05), and no inferential weight is placed on this result. Nevertheless, the descriptive pattern—whereby σ values were numerically larger under the low-predictability condition—is broadly consistent with the proposal of Matzke and Wagenmakers [34] that σ indexes the consistency and stability of cognitive processing; under reduced match quality, greater attentional modulation during integration may contribute to increased processing variability. Whether this pattern reflects a genuine sub-mechanism or constitutes sampling variation cannot be determined from the present data alone and should be treated with caution pending replication.

The τ parameter showed no significant effect in either W4 or W5, suggesting that controlled processing may recede along the success route. Federmeier [3] described a possible neural basis for this transition mechanism: predictive pre-activation reduces the neural and cognitive resources subsequently required for lexical processing, such that when the target word is highly congruent with the prediction, its neural representation is already in a partially activated state, which renders the recognition process more efficient. Accordingly, the success route may reflect the downstream benefit of prior controlled processing: suggesting that greater the controlled resources allocated during Stage 1 may lead to more efficient automatic processing at Stage 2. This parameter pattern could be viewed as a characteristic signature of the success route, potentially reflecting the cognitive advantage of low processing load and fluent forward processing when prediction succeeds. This pattern stands in sharp contrast to Stage 1 and Stage 3, both of which are dominated by τ, as summarised in Table 5.

Put simply, successful prediction appears to convert a potentially effortful recognition process into one that feels seamless—the controlled investment of Stage 1 may be repaid here as automatic processing gain.

#### 4.1.3. Stage 3: Conflict Resolution (Failure Route, W3 and W6)

Prediction failure does not merely slow reading down; it triggers a qualitatively different mode of processing altogether—one in which τ re-emerges at its largest magnitude in the entire study, and does so in the positive direction, suggesting the cost of active, effortful repair.

The conflict resolution stage constitutes an independent repair pathway following unsuccessful expectation verification, encompassing two measurement windows: W6 (target word region TRT) and W3 (pre-target region TRT). In W6, the significant positive τ effect is consistent with lexical activation competition: following verification failure, the pre-activated lexical representation and the actual input may simultaneously compete for activation, with multiple candidate representations activated in parallel. In W3, the significant positive τ effect could be interpreted as reflecting cognitive control-mediated contextual reanalysis: the unresolved competition at the target word may drive regressive eye movements back to the pre-target region, where cognitive control mechanisms potentially engage in top-down reanalysis of the prior context. Together, W6 and W3 form a two-step sequential conflict resolution process.

The predominance of τ at this stage is consistent with the reinstatement of controlled processing following unsuccessful verification. The τ parameter in W6 yielded the largest predictability effect observed in the present study (+19.81 ms, *p* = 0.001), which could reflect the cumulative processing cost incurred during activation competition. Botvinick et al.’s [35] conflict monitoring theory offers one possible framework for this transition: when competing representational activations arise during automatic processing, the conflict monitoring system in the anterior cingulate cortex (ACC) may detect the conflict signal and trigger the prefrontal control system to enhance the target representation and suppress competing representations in a top-down manner. The activation competition in the target word region may drive regressive fixations back to the pre-target region for contextual reanalysis, manifesting as a significant positive τ effect in the total reading time measure of W3. Together, W6 and W3 provide complementary behavioural evidence broadly consistent with a conflict resolution process.

A mismatch between prediction and input renders automatic matching incomplete, potentially prompting a shift to controlled conflict resolution processing. Schneider and Shiffrin [28] characterised the cognitive cost of this transition: whereas automatic processing is fast, parallel, and resource-efficient, the takeover by controlled processing imposes a mode shift to serial, resource-intensive operation. Under this account, the lexical representation pre-activated through controlled processing in Stage 1 not only fails to yield the automatic processing advantage characteristic of Stage 2 but may itself become a source of activation competition owing to its mismatch with the actual input.

Notably, the τ parameter was larger under the low-predictability condition than under the high-predictability condition in both W6 and W3, resulting in an asymmetric pattern of resource allocation. One possible account of this asymmetry is as follows. Under the high-predictability condition, substantial prior resource investment may have constructed a single, strongly dominant candidate representation; the competitive structure would therefore be concentrated around a single dominant candidate, from which competing representations could be rapidly suppressed. Under the low-predictability condition, limited resource investment may have failed to establish a dominant prediction, allowing multiple candidate lexical items to be activated in parallel and to mutually inhibit one another; the resulting competitive structure would be more diffuse, with no single dominant candidate to anchor suppression, thereby increasing the overall cost of conflict resolution. These considerations suggest that the cost of conflict resolution may be determined by the degree of convergence in lexical activation competition, rather than by the strength of the prior prediction per se, though this interpretation remains to be tested directly.

The conflict resolution mechanism is broadly consistent with converging evidence across multiple levels of analysis. Brothers et al. [36] provided evidence that a domain-general conflict monitoring system may account for both neural and behavioural indices of error processing in language comprehension. Clifton et al. [20] suggested that TRT captures the cumulative processing load associated with both lexical identification and sentence-level integration. Frazier and Rayner [37] and Liversedge et al. [38] respectively reported that the repair process following verification failure tends to manifest in late-stage eye-movement measures as prolonged fixation durations and increased regressive eye movements—a pattern broadly consistent with the effect patterns observed in W6 and W3.

It should be noted that the longer fixation durations and positive τ effects observed in the pre-target and target regions could, in principle, partially reflect general increases in reading difficulty rather than prediction-specific mechanisms. These interpretations therefore remain inferential, and alternative accounts cannot be ruled out on the basis of behavioural data alone. Future research employing concurrent ERP or fMRI measurement could help disentangle these accounts by providing direct neural indices of lexical activation competition and conflict monitoring processes.

### 4.2. Integration with Existing Theoretical Frameworks

The present study aims to extend predictive coding theory along four key mechanistic dimensions. First, the expectation construction mechanism (W1–W2) refines the assumption that stronger predictions necessarily require greater top-down resource investment: the data suggest that only when contextual constraints are sufficiently strong might the system commit substantial resources to expectation construction. Second, the rapid automatic integration following successful prediction (W4–W5) is supported by significant μ effects alongside a non-significant σ and τ effects, providing quantitative evidence consistent with the proposition that processing efficiency increases when predictions are accurate. Third, regarding the conflict resolution mechanism following prediction mismatch (W6 → W3): verification failure may first elicit parallel activation competition among multiple candidate lexical items in the target word region (W6), which is accompanied by regressive eye movements back to the pre-target region for contextual reanalysis (W3), thereby potentially specifying how error correction might be behaviourally instantiated within the predictive coding framework. Fourth, concerning the cross-stage dynamic regulation mechanism: the τ effect reverses in direction from negative in W1–W2 to positive in W6 → W3, which may reflect the predictive coding system’s capacity for dynamic regulation across stages and hierarchical levels—the high efficiency of the construction stage may reflect the concerted activation of predictions across multiple levels, whereas the elevated cost following mismatch likely arises from the combined demands of lexical activation competition and conflict resolution.

Kuperberg and Jaeger [4], within a language comprehension framework, distinguished between an automatic associative prediction route and a resource-consuming generative prediction route, providing a theoretical framework that aligns well with the dynamic dissociation between the success route (rapid verification and integration, potentially indexed by μ and σ) and the conflict resolution route (controlled reanalysis, potentially indexed by τ) proposed in the present study. The successful verification route (W4–W5) appears to correspond to the fast, direct lexical pathway, characterised by automatic verification and integration (indexed by μ and σ) with minimal cognitive control demands; the conflict resolution route (W6 → W3) aligns with the slow, cognitive-control pathway, characterised by controlled reanalysis (indexed by τ), and suggests a potential two-stage sequence of lexical activation competition followed by regressive contextual reanalysis. In contrast to the static route distinction assumed in conventional dual-route theories, the present model—through the systematic comparison of μ, σ, and τ parameter effects—points toward both the condition-dependent nature of route selection and the temporal characteristics of dynamic route switching, thus providing quantitative evidence that further informs dual-route theory beyond what static route distinctions alone can offer.

### 4.3. Limitations

Several limitations of the present study should be acknowledged when interpreting the findings. First, it bears emphasising that the μ, σ, and τ parameters of the Ex-Gaussian distribution are statistical descriptors of response time distributions, not direct measures of latent cognitive processes. Their interpretation as indices of specific mechanisms—baseline processing speed, processing variability, and cognitive control, respectively—is theoretically motivated and consistent with prior empirical work (e.g., ref. [9]) but remains inferential rather than directly demonstrated. Second, the three-stage model (expectation generation → verification and integration → conflict resolution) is itself an inferential construct, derived from converging patterns across eye-tracking measures and Ex-Gaussian parameters. It should be understood as a theoretically motivated organisational framework rather than a directly observed cognitive architecture. Third, two features of the experimental design may have introduced item-related or repeated-exposure effects that warrant consideration. Each target word appeared in both predictability conditions and was encountered twice by every participant in the fully within-participant design; although trial order was randomised independently for each participant, the possibility of carry-over effects across conditions cannot be entirely excluded. Fourth, the present findings are specific to native Mandarin-speaking adult readers engaged in silent sentence reading, and generalisation of the three-stage model to other writing systems, populations, or reading modes should be made with caution.

## 5. Conclusions

The present study was motivated by a fundamental limitation in existing research on predictive reading: conventional mean-based analyses treat the predictability contrast as a binary distinction and cannot decompose the distinct cognitive sub-mechanisms underlying predictability effects or reveal their temporal ordering. By applying Ex-Gaussian distributional analysis to eye-movement data from Chinese sentence reading, the three-stage model derived here demonstrates that predictive reading is not a unitary process. In the expectation generation stage, a negative τ effect may reflect efficient top-down expectation construction facilitated by strong contextual constraints. In the verification and integration stage, significant μ and σ effects—without any τ effect—indicate fluent, automatic processing under successful prediction. In the conflict resolution stage, a positive τ effect captures the controlled processing demands triggered by prediction failure. The sign reversal of τ across stages reveals an asymmetric pattern of cognitive resource allocation that mean-based approaches cannot detect.

These findings advance the debate on automaticity versus controlled processing [3,4,5] by demonstrating that the two mechanisms are not mutually exclusive but are differentially recruited across successive processing stages—a dynamic that existing theoretical frameworks [6,7,8] have not previously specified at this level of temporal resolution. Methodologically, the study demonstrates that Ex-Gaussian decomposition offers a level of analytical sensitivity unavailable to conventional analyses, extending its scope as a tool for reading research beyond its established applications in word frequency and syntactic complexity.

Future research should pursue several directions. First, replication using ERP or MEG measures would provide converging neural evidence for the proposed stage–parameter mappings and could help disentangle predictability-specific mechanisms from more general effects of processing difficulty. Second, future research combining Ex-Gaussian analysis with neuroimaging or computational modelling could provide more direct evidence for the parameter–mechanism mappings proposed here. Third, the comprehension-based exclusion criterion used in this study ensures data quality but may limit the generalisability of the findings to populations of highly attentive adult native readers. Whether the proposed three-stage cognitive model applies to readers with lower comprehension efficiency or clinical populations (e.g., individuals with dyslexia) remains an open question; accordingly, future studies could adopt a between-group design comparing readers who differ systematically in reading comprehension ability, in order to examine whether the τ-dominated conflict resolution stage is disproportionately affected in less proficient readers. Fourth, extension to second-language readers and children would test whether the three-stage architecture emerges across different stages of reading development and under conditions of non-native language processing and would help to establish the boundary conditions of the model proposed here.

## Figures and Tables

**Table 1:** Sample stimulus sentences for the high- and low-predictability conditions.

Condition	Sample Sentence	Target Word
High predictability	经过多轮学习，冯埼玉也没有掌握训练技巧与方法。 (Despite repeated coaching, Feng Qiyu still failed to master the core techniques of training.)	训练 (training)
Low predictability	全红婵能取得冠军与她平时的刻苦训练是分不开的。 (Quan Hongchan’s championship victory owes much to her dedicated daily training.)	训练 (training)

Note. Original stimuli were presented in Chinese. English translations are provided in parentheses for reference only. The Chinese target word is shown in its original sentential position.

**Table 2:** Pretarget word properties by predictability condition.

Condition	Word Frequency	Character Frequency		Stroke Count	
		*n* − 2	*n* − 1	*n* − 2	*n* − 1
High predictability	48.62 (52.58)	1576 (3227)	7892 (13,839)	7.80 (2.94)	7.83 (2.75)
Low predictability	40.87 (45.36)	1343 (1893)	6958 (12,201)	8.09 (3.23)	7.76 (2.34)

Note. *n* refers to the first character of the target word; *n* − 1 and *n* − 2 denote the second and first characters of the pretarget word, respectively. Character frequency and word frequency were expressed in occurrences per million; values in parentheses are standard deviations. This reporting format was used in all subsequent tables.

**Table 3:** Eye-movement measures (ms) for the pre-target and target word regions under high- and low-predictability conditions.

Measure	Pre-Target Region		Target Word Region	
	High Predictability	Low Predictability	High Predictability	Low Predictability
FFD	209.65 (34.89)	206.51 (33.25)	215.90 (30.86)	222.30 (35.92)
GD	218.08 (37.24)	213.36 (35.52)	225.76 (34.59)	233.48 (39.92)
TRT	283.69 (52.30)	295.98 (64.51)	281.81 (55.93)	305.53 (65.17)

Note. FFD = first fixation duration; GD = gaze duration; TRT = total reading time. Values are means (standard deviations).

**Table 4:** Ex-Gaussian distribution parameter estimates and inferential test results under high- and low-predictability conditions.

Region	Window	Measure	Parameter	High Predictability (H)	Low Predictability (L)	Difference (L − H)	*t*	*p*	Sig.
Pre-target	W1	FFD	μ	153.83	156.14	+2.31	0.745	0.463	ns
			σ	39.66	40.38	+0.72	0.306	0.762	ns
			τ	56.29	49.86	−6.43	−1.932	0.064	ns
	W2	GD	μ	150.04	153.02	+2.98	1.107	0.278	ns
			σ	34.91	36.88	+1.97	0.702	0.488	ns
			τ	69.21	61.34	−7.87	−2.262	0.031	*
	W3	TRT	μ	136.49	136.30	−0.19	−0.079	0.937	ns
			σ	30.13	30.89	+0.76	0.263	0.793	ns
			τ	147.18	161.17	+13.99	2.377	0.022	*
Target word	W4	FFD	μ	152.77	161.58	+8.81	2.466	0.020	*
			σ	43.57	46.26	+2.69	1.041	0.307	ns
			τ	61.03	59.29	−1.74	−0.484	0.632	ns
	W5		μ	151.25	158.93	+7.69	3.313	0.002	**
		GD	σ	39.43	43.71	+4.28	1.928	0.063	ns
			τ	74.71	74.73	+0.03	0.008	0.993	ns
	W6		μ	146.21	150.13	+3.92	1.208	0.236	ns
		TRT	σ	39.49	38.75	−0.74	−0.366	0.717	ns
			τ	135.60	155.41	+19.81	3.757	0.001	***

Note. ns = not significant; * *p* < 0.05; ** *p* < 0.01; *** *p* < 0.001. Six measurement windows are defined for descriptive convenience: W1–W3 correspond to the early, intermediate, and late temporal windows of the pre-target region (FFD, GD, and TRT, respectively); W4–W6 correspond to the early, intermediate, and late temporal windows of the target word region (FFD, GD, and TRT, respectively). Difference scores represent Low Predictability minus High Predictability (L − H).

**Table 5:** Architecture of the integrated cognitive model of predictive reading.

Cognitive Stage	Window	Eye-Movement Measure	Dominant Mechanism	Cognitive Process	Effect Signature	Mechanistic Description
Stage 1: Expectation Generation (Pre-target Region)	W1	Pre-target FFD	Constraint-driven resource deployment	Automatic cost–benefit evaluation; initiation of resource allocation	τ difference non-significant (negative direction)	Preliminary contextual cues immediately trigger implicit evaluation; resource allocation commences
	W2	Pre-target GD	Predictive pre-activation under contextual constraint	Deepened resource allocation; completion of expectation construction	τ difference significant (negative direction)	High-constraint context increases resource investment; low-constraint context reduces resource investment
Stage 2: Verification and Integration (Success Route)	W4	Target FFD	Automatic prediction verification	Rapid matching	μ difference significant	Automatic verification of prediction–input match
	W5	Target GD	Sustained lexical matching; integration variability modulation	Stable processing	μ difference significant; σ difference non-significant	Integration following successful verification
Stage 3: Conflict Resolution (Failure Route)	W6	Target TRT	Lexical activation competition	Activation competition	τ difference highly significant (positive direction)	Multiple candidate representations activated in parallel, generating competition
	W3	Pre-target TRT	Cognitive control-mediated contextual reanalysis	Contextual reanalysis	τ difference significant (positive direction)	Regressive fixations drive contextual reanalysis of pre-target region

Note. FFD = first fixation duration; GD = gaze duration; TRT = total reading time. Effect direction is indicated in parentheses: negative = shorter durations under high-predictability condition; positive = longer durations under low-predictability condition. Significant: *p* < 0.05; highly significant: *p* < 0.01.

## Data Availability

The data that support the findings of this study are available from the corresponding author upon reasonable request.

## Abbreviations

The following abbreviations are used in this manuscript:

FFD	First Fixation Duration
GD	Gaze Duration
TRT	Total Reading Time
ROIs	Regions of Interest
